# Transferable Plasmids of *Salmonella enterica* Associated With Antibiotic Resistance Genes

**DOI:** 10.3389/fmicb.2020.562181

**Published:** 2020-10-08

**Authors:** Elizabeth A. McMillan, Charlene R. Jackson, Jonathan G. Frye

**Affiliations:** Bacterial Epidemiology and Antimicrobial Resistance Research Unit, U.S. National Poultry Research Center, Agricultural Research Service, U.S. Department of Agriculture, Athens, GA, United States

**Keywords:** *Salmonella*, plasmids, conjugation, antibiotic resistance, horizontal gene transfer

## Abstract

*Salmonella enterica* is a common foodborne illness in the United States and globally. An increasing number of *Salmonella* infections are resistant to antibiotics, and many of the genes responsible for those resistances are carried by plasmids. Plasmids are important mediators of horizontal gene exchange, which could potentially increase the spread of antibiotic resistance (AR) genes. Twenty-eight different incompatibility groups of plasmids have been described in Enterobacteriaceae. Incompatibility groups differ in their accessory gene content, replication mechanisms, and their associations with *Salmonella* serotypes and animal sources. Plasmids also differ in their ability to conjugate or be mobilized, essential genes, and conditions required for transfer. It is important to understand the differences in gene content and transfer mechanisms to accurately determine the impact of plasmids on the dissemination and persistence of antibiotic resistance genes. This review will cover the most common plasmid incompatibility groups present in *S. enterica* with a focus on the transfer mechanisms and associated antibiotic resistance genes.

## Introduction

Non-typhoidal *Salmonella enterica* (NTS) is a leading cause of bacterial foodborne illness, causing over one million infections per year in the United States and more than 93 million globally (Majowicz et al., 2010; Scallan et al., 2011). Salmonellosis generally presents as mild to severe gastroenteritis within 72 h of ingestion of contaminated food or water. Most illnesses are self-limiting, and otherwise healthy individuals usually recover within seven days of symptom onset without antibiotics (Kurtz et al., 2017). However, immunocompromised patients and invasive infections may require antibiotic treatment (Crump et al., 2015). Infections are usually treated with sulfamethoxazole/trimethoprim, ciprofloxacin, or cephalosporins, but resistance to these and other antibiotics has been increasing in *Salmonella* since the 1980s (Theilman and Guerrant, 2004; Crump et al., 2015). The Centers for Disease Control and Prevention (CDC) predicted that 16% of NTS infections in the United States between 2015 and 2018 were resistant to one antibiotic or more (CDC, 2019).

One factor influencing the spread of antibiotic resistance (AR) is horizontal gene transfer (HGT). Mobile genetic elements, including plasmids, phage, and transposons, can facilitate HGT *via* conjugation, transduction, and transformation, respectively (Mazel and Davies, 1999; Frost et al., 2005). Plasmids specifically have featured prominently as agents of HGT associated with AR in Enterobacteriaceae, including *Salmonella* (Carattoli, 2003; Gillings, 2014; Brown-Jaque et al., 2015). In the United States, AR *Salmonella* infections are increasing, highlighting the need for tracking plasmids containing AR genes (CDC, 2019; Tack et al., 2020).

Conjugative plasmids are self-transmissible, giving them the potential to increase the spread of AR genes. The essential components for conjugation are: the origin of transfer (*ori*T), MOB genes, and the mate-pair formation (MPF) genes (Smillie et al., 2010; Banuelos-Vazquez et al., 2017). The MOB genes process the DNA being replicated and transferred to the new cell. The MPF complex forms a channel between the two cells for the DNA to travel through. This protein complex is a subfamily of Type-4 secretion system. However, the genes encoding those proteins vary by plasmid type (Smillie et al., 2010; Wallden et al., 2010; Li et al., 2019). Combined, the MOB and mate-pair genes are sometimes called the *tra* genes because individual genes are often named *tra*.

In order for conjugation to occur, the relaxasome, a protein complex responsible for processing plasmid DNA to prepare it for transfer, must form at the *ori*T (Lawley et al., 2003b; Wong et al., 2012; Waksman, 2019). In the presence of the mate-pair structures, the relaxase then nicks the plasmid DNA and guides it through the translocation channel encoded by the MPF genes (Alvarez-Martinez and Christie, 2009; Ilangovan et al., 2015). However, not all mobile plasmids are conjugative. Non-conjugative plasmids must contain an *ori*T in order for the plasmid to be mobilized, but require a “helper plasmid” that possesses all the necessary MPF genes, which mobilizable plasmids utilize to complete the conjugation process (Loftie-Eaton and Rawlings, 2012). Mobilizable plasmids generally also contain a relaxase. There are eight families of relaxase genes: MOB_B_, MOB_C_, MOB_F_, MOB_H_, MOB_P_, MOB_Q_, MOB_T_, and MOB_V_ (Francia et al., 2004; Garcillan-Barcia et al., 2009; Guglielmini et al., 2011).

Plasmids are typed based on incompatibility with other plasmids, which are defined as the inability of two plasmids to be maintained together in the same cell line (Novick et al., 1976). Replication genes can differ by incompatibility group. These genes can be detected with PCR for plasmid typing (Carattoli et al., 2005). Relaxase genes can also be used for typing by a similar process (Compain et al., 2014). Twenty-eight incompatibility groups have been isolated in Enterobacteriaceae (Carattoli, 2009; Rozwandowicz et al., 2018). Plasmids of different incompatibility groups differ in their host range, size, and transfer mechanism (Rozwandowicz et al., 2018). They can also differ by the AR genes they carry.

Despite genetic similarities, other Enterobacteriaceae species like *Escherichia coli*, are not good predictors of AR in *Salmonella*, even from the same sample (Nyirbahizi et al., 2020). Additionally, distribution of plasmid types and accessory genes these plasmids carry can differ in *Salmonella* compared to other Enterobacteriaceae, although estimates of plasmid frequencies can be biased by the tendency to sequence clinically relevant strains from humans and animals (Williams et al., 2013). Further, even though *Salmonella* contain plasmids that can be found in many organisms, serotypes of *Salmonella* differ in the frequencies of plasmids they contain, and new plasmids can emerge within any serotype. While some serotypes are more likely to contain certain plasmids than others, these plasmids can exist in any serotype, and no incompatibility group is confined to a single serotype. As *Salmonella* is an important human pathogen known to develop AR and potentially transfer it between animals and humans, it is recognized globally as a sentinel organism for surveillance programs like the National Antimicrobial Resistance Monitoring System (NARMS). *Salmonella* also contains unique plasmids; therefore, this review will focus on characteristics of the major incompatibility groups found in *Salmonella*, which are summarized in Table 1. Presented examples of serotypes that can contain certain plasmids more frequently than others are summarized in Table 2.

**Table 1:** Summary of features of transferable plasmids associated with *Salmonella enterica*.

Type	Transfer mechanism^*^	Replicon Type^a^	Relaxase Type^b^	Common associated antibiotic resistances^*^,^c^
C	Conjugative	A/C	MOB_H_	Aminoglycosides, tetracyclines, trimethoprim, chloramphenicols, β-lactams, and sulfonamides
F	Conjugative	F, FII, FIA, FIB, and FV	MOB_F_	Aminoglycosides, tetracyclines, trimethoprim, chloramphenicols, β-lactams, sulfonamides, and fluoroquinolones
HI1 and HI2	Conjugative	HI1 or HI2	MOB_H_	Chloramphenicols, β-lactams, sulfonamides, aminoglycosides, tetracyclines, and fluoroquinolones
I1	Conjugative	I1	MOB_P_	Chloramphenicols, β-lactams, sulfonamides, aminoglycosides, and tetracyclines
N	Conjugative	N	MOB_F_	Fluoroquinolones
Q1	Mobilizable	N/A	MOB_Q_ (MOB_P_)	Aminoglycosides, tetracyclines, and sulfonamides
R	Unknown	R	N/A	Aminoglycosides, tetracyclines, trimethoprim, chloramphenicols, β-lactams, sulfonamides, and fluoroquinolones
X	Conjugative (repressed)	X(1–6)	MOB_P_	Aminoglycosides, β-lactams, and quinolones

* See text for references.

a From PCR Based replicon typing (Carattoli et al., 2005).

b From (Garcillan-Barcia et al., 2009).

c Not comprehensive.

**Table 2:** Incompatibility groups with examples of associated *Salmonella* serotypes.

Plasmid incompatibility group	*Salmonella* serotype^*^	Associated genes
C	Dublin Newport	*str*AB (aminoglycosides), *sul*2 (sulfonamides), *tet*AR (tetracycline), *bla_CMY-2_* (β-lactams), and *flo*R (chloramphenicol)^b^
F	Abortusequi Abortusovis Choleraesuis Enteriditis Gallinarum Sendai Typhimurium	*spv* operon
	Kentucky	Avian pathogenic *Escherichia coli* virulence plasmid
X1/F mosaic	Dublin^a^	*spv* operon
FIB/I1/P mosaic	Infantis	*bla_CTX-M-65_* and virulence genes

* See text for references.

a (Platt et al., 1988).

b Other antibiotic resistance (AR) genes can be present.

## IncC

One of the most well-studied incompatibility groups found in *Salmonella* is IncA/C. Initial studies indicated that the IncA and IncC groups were closely related and exhibited entry exclusion toward each other so the groups were combined (Hedges, 1974). However, it was confirmed that IncA and IncC plasmids are compatible, and the use of the IncA/C term should be discontinued (Ambrose et al., 2018a). Since IncA/C has been grouped together historically, plasmids that have not been reclassified will be designated as IncA/C because PCR is not a strong enough tool to differentiate them (Ambrose et al., 2018a).

IncC plasmids are large, low copy number, broad host range, and frequently contain AR genes (Harmer and Hall, 2015). In contrast, only five IncA plasmids have been isolated (none from *Salmonella*; Ambrose et al., 2018a). Most plasmids are conjugative, but co-carriage of another large plasmid may increase the transfer rate (Han et al., 2018). IncC plasmids encode a MOB_H_ relaxase (Garcillan-Barcia et al., 2009). Essentials for IncC plasmid transfer are the AcaC and AcaD proteins, which serve as master activators for the *tra* genes and at least two chromosomally encoded genomic islands: *Salmonella* genomic island 1 (SGI1) and a mobilizable chromosomal element in *Vibrio mimicus* (Carraro et al., 2014a). The genes *tra*N, *tra*H, and *tra*G have also been determined to be essential for IncC transfer and can facilitate the mobilization of SGI1 (Carraro et al., 2017). IncC plasmids contain other *tra* genes, but their functions have not been confirmed experimentally (Harmer and Hall, 2015; Ambrose et al., 2018b). The relaxase-like gene *mob*I is also required for IncC transfer (Carraro et al., 2014b). While the biochemical function of the gene is unknown, it is located immediately downstream of the *ori*T and essential for plasmid transfer, but unrelated to SGI1 mobilization (Hegyi et al., 2017). In IncC plasmids, the gene *eex*C facilitates entry exclusion toward IncA plasmids as well as undefined incompatibility groups by recognizing *tra*G (Humbert et al., 2019).

IncA/C plasmids have been isolated in *Salmonella* that contain up to 10 AR genes for more than five classes of antibiotics. The most common AR genes carried by IncA/C are *str*AB (aminoglycosides), *sul*2 (sulfonamides), *tet*AR (tetracycline), *bla*_CMY-2_ (β-lactams), and *flo*R (chloramphenicols). Other genes for resistance to aminoglycosides, tetracyclines, trimethoprim, chloramphenicols, and cephalosporins have also been identified (Welch et al., 2007; Hoffmann et al., 2017; Cao et al., 2018). In *Salmonella* isolated in the United States, IncA/C plasmids have been found in several different serotypes and animal commodities, especially cattle (Lindsey et al., 2009; Folster et al., 2017; Mollenkopf et al., 2017). Notably, IncA/C plasmids have been found in many *Salmonella* Newport isolates (Table 2; Cao et al., 2015). *Salmonella* Newport has been isolated from cattle containing IncC plasmids with AR genes for at least five classes of antibiotics (Cao et al., 2018). Isolates in a 2017 study of human and dairy cattle associated *Salmonella* of serotypes Dublin, Newport, and Typhimurium showed that all Dublin and Newport isolates tested contained *sul*2, *str*A, *str*B, *tet*A, and *bla*_CMY-2_. More than 75% of all isolates tested also contained *flo*R, and one-third of the isolates contained an IncA/C plasmid (Carroll et al., 2017). However, more investigation is needed to explain why these serotypes are more likely to contain IncC plasmids.

## IncF

IncF plasmids are large (>80 kb), low-copy number, and host restricted to Enterobacteriaceae. They are important because many virulence-associated plasmids, those that allow a host bacterium to cause a more virulent infection, of *Salmonella* are IncF (Silva et al., 2017; Rozwandowicz et al., 2018). An example of virulence genes in *Salmonella* plasmids is the *spv* genes (Boyd and Hartl, 1998). The *spv* operon increases the invasive nature of *Salmonella* and host cytotoxicity (Guiney and Fierer, 2011). Serotypes Typhimurium, Dublin, Enteritidis, Choleraesuis, Abortusovis, Abortusequi, Gallinarum, and Sendai usually contain a virulence plasmid (Table 2; Boyd and Hartl, 1998; Uzzau et al., 2000; Anzai et al., 2005; Guiney and Fierer, 2011). *Salmonella* Kentucky isolates have been identified containing an IncF virulence plasmid acquired from an avian pathogenic *E. coli* (APEC) strain that may confer an advantage in an avian host (Table 2; Johnson et al., 2010).

The conjugation systems of IncF plasmids are diverse. They can be categorized into five classes based on the genes regulating the expression of the transfer genes; only two of the groups, one and three, have been seen in *Salmonella* (Fernandez-Lopez et al., 2016). The transfer pilus is one of the defining features of IncF plasmids as they are similar across all IncF plasmids but distinct from other incompatibility groups (Lawley et al., 2003b; Fernandez-Lopez et al., 2016). Relaxase genes carried by IncF plasmids are classified as MOB_F_ (subtype MOB_F12_; Garcillan-Barcia et al., 2009). In contrast to other incompatibility groups, IncF plasmids can carry multiple types of replicon associated genes, such as FIA, FII, or FIB (Villa et al., 2010).

IncF plasmids have been isolated that contain AR genes. IncF plasmids in *Salmonella* isolated in China carried fluoroquinolone resistance genes (Chen et al., 2018). IncF plasmids isolated from *Salmonella* I 4,[5],12:i:-, the monophasic variant of Typhimurium, carried *bla*_TEM-1_, *cml*A (chloramphenicols), and an integron containing *drf*A (trimethoprim), *aad*A1 and *aad*A2 (aminoglycosides), and *sul*3 (Garcia et al., 2014). In *Salmonella* isolated in the United States, IncF plasmids have been associated with *str*AB, *tet*A, *tet*C, *tet*D, *aph*A (aminoglycosides), and *sul*2 (Han et al., 2012; McMillan et al., 2019).

## IncHI

First described in *Salmonella* Typhi in 1972, IncHI plasmids are classified into three groups: HI1, HI2, and HI3 (Anderson and Smith, 1972; Rozwandowicz et al., 2018). IncHI plasmids are generally conjugative and very large, containing up to 300,000 base pairs (Gilmour et al., 2004). HI1 and HI2 plasmids have a well-conserved backbone structure with regions of variation (Holt et al., 2007). However, HI1 plasmids have two distinct lineages based on the presence and absence of accessory gene regions (Cain and Hall, 2013). HI1 and HI2 plasmids are often isolated in *Salmonella*, but not host-restricted (Maher and Taylor, 1993; Holt et al., 2007; Chen et al., 2016).

Conjugation in IncHI plasmids is temperature-dependent. Conjugation is optimized at 27°C, but repressed at 37°C, the normal body temperature for warm blooded hosts of *Salmonella* (Maher et al., 1993). Relaxase genes of both HI1 and HI2 plasmids are classified as MOB_H_ subtype MOB_H11_ (Garcillan-Barcia et al., 2009). In HI1 and HI2 plasmids, the transfer gene region is split into two locations, Tra1 and Tra2 (Taylor et al., 1984). Tra1 contains 14 genes, including five MPF genes and five non-essential genes (Lawley et al., 2002). Tra2 contains 28 genes, including four involved in partitioning the plasmid between two cells, 11 MPF genes, 10 non-essential genes, and two that regulate transfer frequency (Lawley et al., 2003a). A novel Ig-containing protein (contains an imunoglobulin-like motif), RSP, is also required for conjugation (Huttener et al., 2019).

IncHI plasmids have been isolated that contain heavy metal resistance genes as well as the *tet*B gene of the transposon Tn10 (Gilmour et al., 2004; Cain and Hall, 2012a,b). In addition to *tet*B, plasmids are also associated with resistance genes for streptomycin and sulfonamides (Rozwandowicz et al., 2018). In the globally circulating MDR *Salmonella* Typhi strain, HI1 plasmids carry *cat* (chloramphenicol), *str*AB, *tet*AR, *sul*2, and *bla*_TEM-1_ (Phan et al., 2009). IncHI plasmids have also been associated with *qnr* genes (fluroquinolones) and ESBL genes (Chen et al., 2016). Genes conferring colistin resistance have been found on HI2 plasmids in *Salmonella* in several countries, including Canada and Portugal (Figueiredo et al., 2016; Mulvey et al., 2018; Lima et al., 2019).

## IncI1

IncI1 plasmids are large, conjugative, and host-limited to Enterobacteriaceae (Carattoli et al., 2018). The prototypical plasmid, R64, was isolated from *Salmonella* Typhimurium during a study examining the differences in plasmids before they were classified by incompatibility (Meynell and Datta, 1966; Sampei et al., 2010). IncI1 genetic structure is well-conserved, except for the accessory gene region, which varies by individual plasmid (Carattoli et al., 2018). The transfer region is approximately 54 kb and contains at least 49 genes, up to 35 of which are essential, depending on the plasmid (Komano et al., 1990, 2000). IncI1 contain a MOB_P_ relaxase, generally subtype MOB_P12_ (Garcillan-Barcia et al., 2009). Cefotaxime (126 mg/L, half the minimum inhibitory concentration) can cause an upregulation of transfer genes in IncI1 plasmids that contain cefotaxime resistance genes (Moller et al., 2017).

While genetically similar to the transfer region of IncF plasmids, IncI1 plasmids exhibit distinct differences: two types of pili and a unique shufflon system. Both pili, one thick and one thin, are required for conjugation on a surface, while only the thin pilus is required for conjugation in broth (Bradley, 1983; Komano et al., 2000). The shufflon system allows for several variants of the *pil*V gene, which encodes adhesins that recognize lipopolysaccharide targets on the cell surface during conjugation (Komano et al., 1986; Sampei et al., 2010). This variation allows IncI1 plasmids to transfer to a broader range of hosts. For example, *E. coli* and *Salmonella* require a different *pil*V variant for transfer (Ishiwa and Komano, 2003; Sekizuka et al., 2017).

IncI1 plasmids are subdivided into hundreds of sequence types with a pMLST scheme based on the *pil*L (pilus biosynthesis), *sog*S (primase), *ard*A (restriction-modification enzyme), *rep*I1 (RNAI), and a region between the *trb*A and *pnd*C genes (Garcia-Fernandez et al., 2008). They are also divided into clonal complexes that contain specific AR genes (Carattoli et al., 2018). IncI1 plasmids play a major role in the dissemination and persistence of β-lactamase genes (Carattoli et al., 2018). The *bla*_CMY-2_ gene is especially prevalent among IncI1 plasmids (Folster et al., 2011). IncI1 plasmids are also frequently implicated as carriers of AR genes in *Salmonella* associated with poultry (Folster et al., 2014). In *Salmonella* Heidelberg, IncI1 plasmids have been associated with an integron containing *aad*A, *aac*(3')-IId (aminoglycosides), and *sul*1, *bla*_TEM-1_, and *tet*A; some isolates were responsible for an outbreak associated with turkey in the United States in 2011 (Folster et al., 2012; Han et al., 2012).

## Other Incompatibility Groups

Other incompatibility groups have been found carrying AR genes in *Salmonella*. IncN plasmids are a small (30–70 kb) broad host range plasmid categorized by a pMLST scheme based on the *rep*N, *kor*A, and *tra*J genes (Garcia-Fernandez et al., 2011; Rozwandowicz et al., 2018). They contain a MOB_F_ relaxase (Garcillan-Barcia et al., 2009). The transfer region of IncN plasmids is split into three discontinuous segments (Winans and Walker, 1985). In *Salmonella*, IncN plasmids have been associated with *qnr* genes (Garcia-Fernandez et al., 2009; Kim et al., 2013).

IncX plasmids range from 30 to 50 kb in size and are divided into six subgroups: X1–X6 (Compain et al., 2014; Rozwandowicz et al., 2018). Although most common in *Salmonella* and *E. coli*, transfer is possible to *Pseudomonas aeruginosa* (Tardif and Grant, 1983; Johnson et al., 2012). Conjugation in IncX plasmids is naturally repressed, but can be derepressed by suspending cells in an ammonium acetate solution (Bradley, 1980). IncX plasmids contain a MOB_P_ relaxase, but the subtype differs by individual plasmid (Garcillan-Barcia et al., 2009). In *Salmonella* isolated in the United States, IncX plasmids have been associated with resistance to β-lactams and aminoglycosides (McMillan et al., 2019). IncX plasmids carrying ESBL and quinolone resistance genes have also been isolated (Rozwandowicz et al., 2018).

## Mobilizable Plasmids

Mobilizable plasmids in *Salmonella* also contain AR genes. IncQ1 plasmids are small (10–12 kb), have a well-conserved structure, and broad host range. They are generally associated with *str*AB, *tet*AR, and *sul*2, although other AR genes have been identified (Poirel et al., 2010; Oliva et al., 2017). IncQ1 plasmids replicate *via* strand-displacement and contain a relaxase gene, *mob*A, fused to a *rep*B primase. The relaxase genes of IncQ1 plasmids are diverse, sometimes only exhibiting 84% homology between different plasmids (Loftie-Eaton and Rawlings, 2012). This could explain why IncQ1 relaxases can be classified as either MOB_Q_ or MOB_P_ (Garcillan-Barcia et al., 2009). IncQ1 plasmids can be mobilized by large plasmids including types F, I1, N, P, W, and X (Willetts and Crowther, 1981; Francia et al., 2004).

IncR plasmids were first described in 2009 in isolates of *Klebsiella* and *Salmonella* Montevideo (Garcia-Fernandez et al., 2009). They are not thought to be conjugative since they do not contain any *tra* genes or a relaxase, but could be mobilizable (Chen et al., 2006; Bielak et al., 2011; Rozwandowicz et al., 2018). Although IncR plasmids are rarely reported, an outbreak strain of *Salmonella* Newport in the United States reported between 2018 and 2019 contained an IncR plasmid carrying genes for resistance to up to five different classes of antibiotics: trimethoprim/sulfamethoxazole, tetracycline, chloramphenicol, β-lactams, and aminoglycosides (Plumb et al., 2019). IncR plasmids have also been associated with resistance to quinolones (Rozwandowicz et al., 2018).

## Discussion

Understanding how plasmids transfer in *Salmonella* is of the utmost importance in determining the risk of the dissemination of AR genes. If a plasmid carrying AR genes is conjugative, genes may have a higher risk of dissemination than those carried by non-conjugative plasmids. Furthermore, if a plasmid is mobilizable, it is important to know if the cell carries a helper plasmid and of what type; conjugative plasmids mobilize non-conjugative plasmids at different rates (Cabezon et al., 1997). However, it is important to remember that presence of conjugation genes does not guarantee conjugation. External conditions influence transfer efficiency; for example, inflammation has been shown to increase rates of transfer for IncI1 plasmids from *Salmonella* to *E. coli* (Stecher et al., 2012).

Plasmids are common in *Salmonella* and differ in their gene content and transfer genes. Tracking these plasmids is important because some can be associated with certain AR genes, like β-lactamases with I1 plasmids (Carattoli et al., 2018). However, as AR genes are frequently associated with transposons, genes are not fixed to specific plasmids, and AR genes can emerge on new plasmids at any time. Additionally, plasmids can contain multiple replicons. One study found that 66% of *Salmonella* plasmids investigated were mosaic, meaning that they contained sequences from multiple different plasmids as a result of recombination; many were mosaics of plasmids from different incompatibility groups (Boyd et al., 1996; Pesesky et al., 2019). Recently, a *Salmonella* Infantis strain containing the mosaic pESI-like plasmid (plasmid for emerging *Salmonella* infantis) carrying *bla*_CTX-M-65_ has emerged in poultry and caused human infections around the world (Table 2; Aviv et al., 2014; Tate et al., 2017; Brown et al., 2018). Although this gene has been isolated on other plasmids in *E. coli*, the gene is only found on the pESI plasmid in *Salmonella* Infantis. This plasmid has contributed to the rise of Infantis as one of the dominant serotypes in poultry in Europe and in the United States, where the prevalence of the serotype increased nearly 70% in the past few years (Tack et al., 2020). In addition to *bla*_CTX-M-65_, this plasmid carries other AR genes and genes that convey advantages over other *Salmonella* within a poultry host.

Plasmids in *Salmonella* may acquire AR genes seemingly at random, but their proliferation in a population is not random. The pESI plasmid is a good example of how a strain with a plasmid containing AR genes can rapidly spread by co-selection. Plasmids in *Salmonella* often carry more than one AR gene, or genes for resistance to heavy metals and/or biocides. In these cases, even if an antibiotic is withdrawn from the environment, other selective pressures encouraging plasmid carriage may remain. Further, plasmids may contain other factors, like fimbriae or nutrient acquisition systems, that give the host bacterium an advantage that further selects for the plasmid. Even without another selecting factor, plasmids may not carry a high fitness cost to the host bacterium. In some cases, AR genes carried by plasmid can have a lower cost of fitness than those carried chromosomally (Vogwill and MacLean, 2014).

Co-selection could also play a part in the associations between serotypes and certain plasmid types, but further investigation is needed to fully understand these correlations. For example, it is unknown why IncC plasmids are more common in serotypes Newport and Dublin. In the United States, these serotypes are also more frequently isolated from cattle sources, but whether the relationship of the plasmid is related to the serotype, the source, or a combination of factors remains unknown. Co-selection could also be responsible for the association of the pESI plasmid with serotype Infantis in poultry but does not explain why the plasmid has not been isolated in any other poultry-related serotypes. In this case, another factor such as a barrier to conjugation must be influencing the serotype association.

Surveillance is necessary, not just of *Salmonella* and other important human and animal pathogens, but of the plasmids they carry. Tracking plasmids and the genes they carry would allow for a better understanding of co-selection of AR genes and the associations of plasmids with *Salmonella* serotypes. Plasmid surveillance will not prevent the spread of AR, but it would provide information for designing mitigation strategies that account for these factors. Further study is needed to assess the contribution of plasmids to the spread of AR in *Salmonella*, both genotypically and phenotypically.

## Author’s Note

The mention of trade names or commercial products in this manuscript is solely for the purpose of providing specific information and does not imply recommendation or endorsement by the U.S. Department of Agriculture. USDA is an equal opportunity provider and employer.

## Author Contributions

EM, CJ, and JF all wrote the manuscript. All authors contributed to the article and approved the submitted version.

### Conflict of Interest

The authors declare that the research was conducted in the absence of any commercial or financial relationships that could be construed as a potential conflict of interest.
